# Emerging trends and research foci of epithelial–mesenchymal transition in gliomas: A scientometric analysis and review

**DOI:** 10.3389/fonc.2022.1015236

**Published:** 2022-10-20

**Authors:** Yang Xing, Minghua He, Zhenjin Su, Feroza Yasinjan, Jiankai Liu, Hong Wang, Jiayue Cui, Xinyu Hong

**Affiliations:** ^1^ Department of Neurosurgery, The First Hospital of Jilin University, Chang Chun, China; ^2^ College of Computer Science and Technology, Jilin University, Chang Chun, China; ^3^ Department of Biochemistry, College of Basic Medical Sciences, Jilin University, Chang Chun, China; ^4^ Cancer Center, The First Hospital of Jilin University, Chang Chun, China; ^5^ Department of Histology and Embryology, College of Basic Medical Sciences, Jilin University, Chang Chun, China

**Keywords:** EMT, gliomas, GBMs, scientometric, visualization, citespace, VOS viewer

## Abstract

**Background:**

Epithelial–mesenchymal transition (EMT) is a key factor in the invasion and migration of glioma cells, and the study of EMT in gliomas has become a hot topic over the past decade. Scientometric analysis is gaining more attention since it can obtain hot topics and emerging trends in a research field. This article analyzed the research related to EMT in gliomas for the first time, including descriptions of research situations, evaluations of research foci, and predictions of emerging trends.

**Methods:**

We searched the topic-related original articles from January 2012 to December 2021 in the Web of Science Core Collection (WoSCC) by using a specific strategy, and a total of 1,217 publications were obtained. The WoS platform, VOS viewer, and CiteSpace were used to analyze the annual distribution of publications and citations, authors and density of keywords, and other analyses including countries, institutions, references, clustering, burst analysis, and the timeline view of keywords.

**Results:**

Scientometric analysis identified that the study of EMT in gliomas has developed fast and received continuous attention in the last decade. Based on the results of data analysis, most publications on the topic came from China, and the United States had the highest betweenness centrality. The top 10 co-cited references revealed the landmark documents that had greatly promoted the development of this field. The major focus is on the cellular and molecular mechanisms of EMT in gliomas, and the therapy related to EMT target and non-coding RNAs has been developing fast in recent years.

**Conclusions:**

This study revealed the intimate connections between EMT and gliomas, and the complex mechanisms regulating EMT in gliomas had been studied widely in the last decade. Exploring the deep mechanisms of EMT in gliomas is the foundation of the targeted inhibitions, which can promote the development of therapies for gliomas.

## 1 Introduction

Glioma is one of the most common intracranial tumors worldwide with the characteristics of high invasion and aggression (1). Gliomas can be classified into four grades, and glioblastoma (GBM; grade IV) is the most common and most malignant (1). An important reason for the high invasion of gliomas is the epithelial–mesenchymal transition (EMT). Glioma cells undergoing EMT become mesenchymal and gain cell invasion and migration by expressing EMT markers including N-cadherin, β-catenin, vimentin, matrix metalloproteinases (MMPs), Twist1/2, Snail/Slug, and Zeb1/2 (2–5). Although EMT is closely related to the invasion, migration, metastasis, and therapeutic resistance of gliomas, the study on EMT in gliomas started rather late, and many unknown issues are yet to be explored (3).

Scientometric analysis uses both quantitative and qualitative methods to understand the knowledge structure and explore research trends (6, 7). It is widely applied in the field of medical science to obtain the current research situations, new developments, and research trends of a specific topic (8, 9).

This study analyzed the relevant publications from Web of Science Core Collection (WoSCC) in the past decade (2012–2021) through scientometric analysis and gave a summary of hot topics and emerging trends in this field. The study could shed light on the importance of EMT in gliomas and could provide new insights for related scientists.

## 2 Materials and methods

### 2.1 Data source and search strategy

The Science Citation Index Expanded (SCIE) in WoSCC was used as the data source. The search strategy was developed according to Medical Subject Headings (MeSH) database (https://www.ncbi.nlm.nih.gov/mesh/) and free words: #1 TS=(“Glioma” OR “Gliomas” OR “Astrocytoma” OR “Astrocytomas” OR “Glioblastoma” OR “Glioblastomas” OR “Ependymoma” OR “Ganglioglioma” OR “Gliosarcoma” OR “Medulloblastoma” OR “Medulloblastoma”) AND #2 TS=(“Epithelial-Mesenchymal Transition” OR “Epithelial-Mesenchymal Transitions” OR “EMT” OR “Epithelial to Mesenchymal Transition”). Document type: Article. Language: English. Publication years: 2012-2021. The searching process was conducted on 17 March 2022, and 1,217 documents were incorporated into this study.

### 2.2 Methodology of data analysis

#### 2.2.1 Web of Science

Web of Science (WoS) is a platform providing both credible global citation data and basic scientometric analysis. WoS was applied to analyze the annual distribution of publications and citations since it provides up-to-date literature data.

#### 2.2.2 CiteSpace (5.8.R3)

A total of 1,217 records were exported in the form of a plain text file from WoSCC to CiteSpace. In addition, 1,216 records were obtained the duplicates were removed. Adjustments of CiteSpace included 1) setting Time Slicing as 2012 Jan–2021 Dec, one year per slice, 2) setting Selection Criteria as Top 50 levels from each slice, and 3) setting Pruning as default except for cluster analysis of keywords. In this study, CiteSpace provided the analysis of researching countries and institutions, references, clustering, burst analysis, and the timeline view of keywords.

#### 2.2.3 VOS viewer (1.6.18)

The records were exported in the form of a tab-delimited file from WoSCC to the VOS viewer. In this study, VOS viewer was applied to analyze researching authors and the density of keywords.

The word co-citation is originally defined as the frequency that two documents are cited together by other documents (10). Co-citation analysis can reveal the similarity of a document with other documents, which means that high co-citations can reflect the importance of documents (or journals and authors) in a way that is more focused on the target topic than high citations (10).

The word cluster means grouping a set of objects in such a way that objects in the same group (called a cluster) are more similar to each other than to those in other groups (11). The cluster keyword is the most central and significant in a cluster.

## 3 Results

### 3.1 Annual distributions of publications and citations

**Figure 1:**
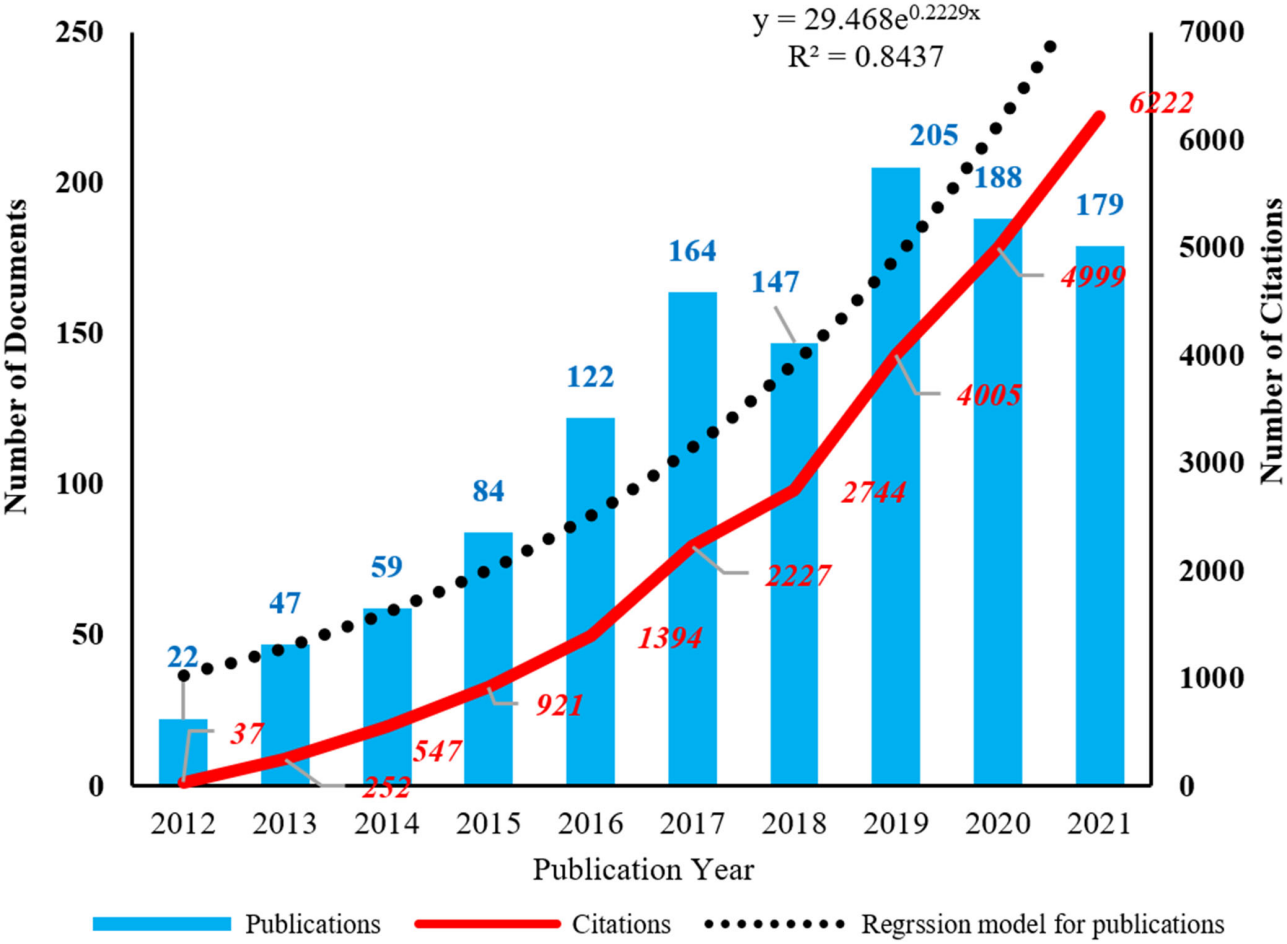
The annual distribution of publications and citations. The corresponding regression model for publications is y = 29.648e^0.2229x^ (R^2^ = 0.8437).

The distribution of publications and citations was obtained from WoS (**Figure 1**). It showed that the publication number continued to increase before 2017 and reached its peak in 2019 with 205 documents. The publication kept relatively stable in 2020 and 2021 with 188 and 179 documents, respectively. The citation number continued to increase rapidly in the last decade. To better understand how publication number changes over time, a regression model y = 29.648e^0.2229x^ (R^2^ = 0.8437) was adopted to fit the graph. In addition, the H index of this field from WoS is 62, meaning that 62 documents had received more than 62 citations. The above analysis showed that the field of EMT in gliomas developed fast and received continuous attention in the last decade and could probably continue to thrive in the future.

### 3.2 Research countries or regions and institutions

**Figure 2:**
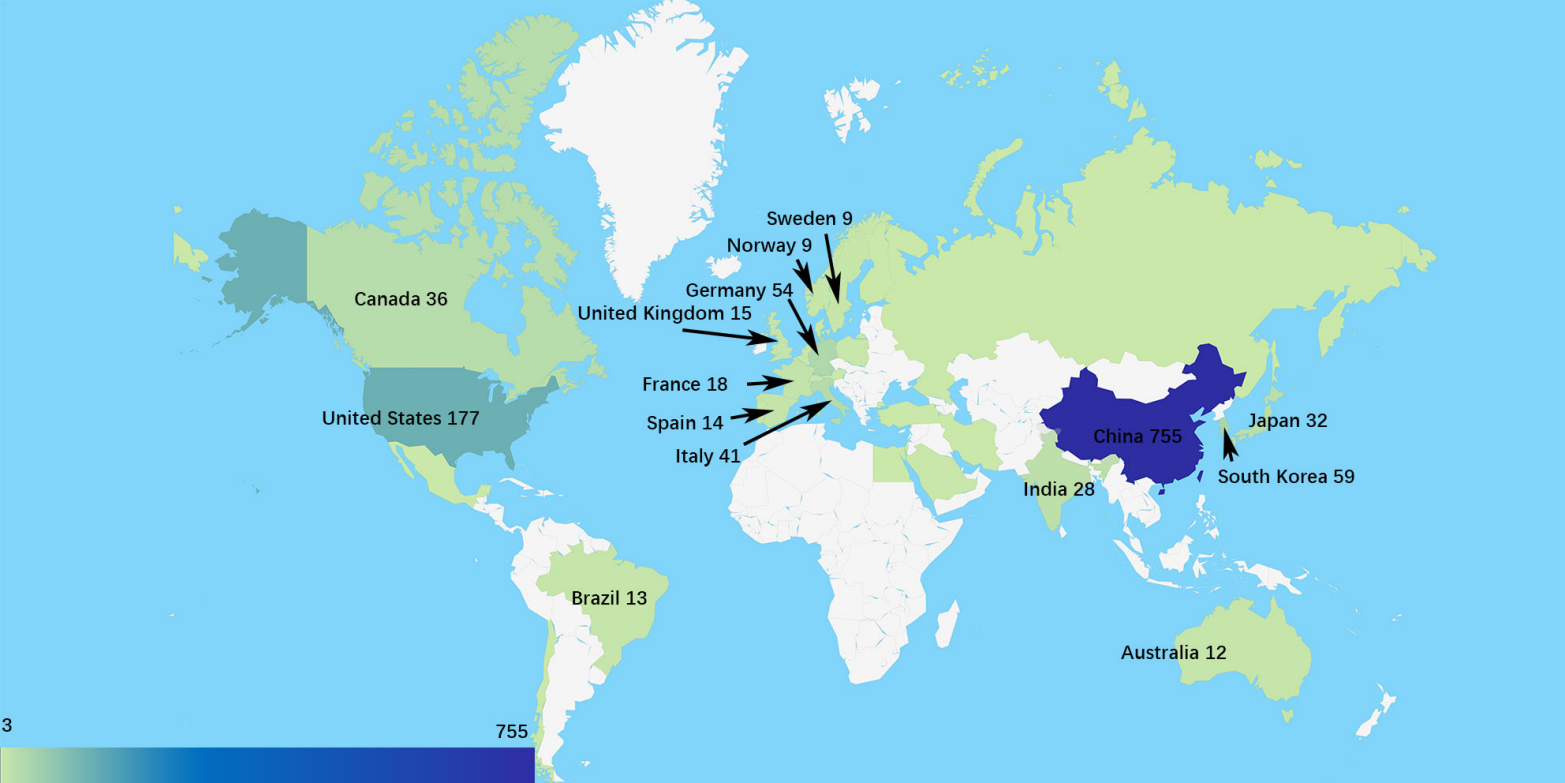
The geographical distribution based on the number of publications.

The data on geographical distribution (**Figure 2**) were from CiteSpace. There were 33 countries or regions owning three or more publications in this field. The scientists from the People’s Republic of China (China) were the most productive, and the Chinese publication percentage of total documents is 62.04%, which is much higher than that of countries like the United States (177, 14.54%), South Korea (59, 4.85%), and Germany (54, 4.44%). Betweenness centrality is the indicator of the importance of a node in co-occurrence networks (7). In this study, the United States had the highest centrality of 0.76. Germany and China ranked second and third with centralities of 0.30 and 0.22, respectively.

**Figure 3:**
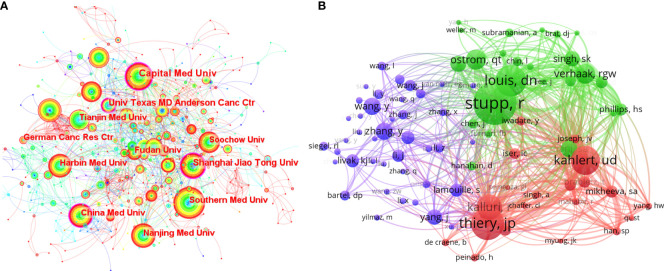
**(A)** The collaborative network of the research institutions. **(B)** The collaborative network of co-cited authors.

The collaborative network of research institutions (**Figure 3A**) was from CiteSpace. A total of 789 institutions participated in the research related to EMT in gliomas, and the most productive 10 institutions were located in China. Southern Medical University ranked first with 47 publications. Analysis of betweenness centralities showed that there were six Chinese institutions and four US institutions on the list of top 10 institutions. Centralities of Capital Medical University (0.19), University of Texas MD Anderson Cancer Center (0.16), China Medical University (0.14), and Shanghai Jiao Tong University (0.12) were over 0.10.

The publication number of countries or institutions indicated that the United States and China were the main research countries in this field. The analysis of centralities showed that the United States was dominant in this field. Capital Medical University in China and the University of Texas MD Anderson Cancer Center in the United States were the dominant institutions.

### 3.3 References

The top 10 co-cited references from CiteSpace are introduced in **Table 1**. There are three articles about Zeb1 (4), Snail (5), and genomic analysis (16) and seven reviews from authoritative journals. These highly co-cited references represented the classical literature in this field. For example, the integrated genomic analysis of GBM (Cancer Cell, 2010) (16) appeared with high co-citations in the two analyses from CiteSpace (**Figure S1**) and VOS viewer (**Figure S2**). Considering the time span of the research on EMT in gliomas, this article may be the triggering factor for the following wide exploration. The review of Kahlert UD (Cancer Letters, 2013) (2) was probably the first comprehensive review of EMT in gliomas, which helped researchers know about EMT in gliomas in a general way. Undoubtedly, these landmark documents have greatly promoted the development of this field.

**Table 1:** The top 10 co-cited documents.

Year	Title	Type	First author	Journal	Focus and main idea	IF (2021)	JCR	Co-citation
2016	The 2016 World Health Organization Classification of Tumors of the Central Nervous System: a summary	Review	Louis DN (1)	*Acta Neuropathological*	A classification system of tumors of the central nervous system in 2016	15.887	Q1	82
2013	Epithelial-to-mesenchymal(-like) transition as a relevant molecular event in malignant gliomas	Review	Kahlert UD (2)	*Cancer Letters*	The earliest review of EMT in gliomas	9.756	Q1	53
2017	The Epithelial-to-Mesenchymal Transition-Like Process in Glioblastoma: An Updated Systematic Review and In Silico Investigation	Review	Iser IC (3)	*Medicinal Research Reviews*	A review of EMT in GBM	12.388	Q1	50
2014	Molecular mechanisms of epithelial–mesenchymal transition	Review	Lamouille S (12)	*Nature Reviews Molecular Cell Biology*	A review of molecular mechanisms in EMT	113.915	Q1	46
2016	Epithelial–mesenchymal transition in glioblastoma progression	Review	Iwadate Y (13)	*Oncology Letters*	Emphasis on EMT-inducing factors that are present in gliomas	3.111	Q3	40
2013	The ZEB1 pathway links glioblastoma initiation, invasion and chemoresistance	Article	Siebzehnrubl FA (4)	*EMBO Molecular Medicine*	The research of relationships between Zeb1 pathway and GBM	14.26	Q1	35
2014	Sustained elevation of Snail promotes glial–mesenchymal transition after irradiation in malignant glioma	Article	Mahabir R (5)	*Neuro-Oncology*	Proposal of glial–mesenchymal transition and how Snail regulates it	13.029	Q1	30
2016	EMT: 2016	Review	Nieto MA (14)	*Cell*	A comprehensive and authoritative review of EMT	66.85	Q1	29
2013	Regulatory networks defining EMT during cancer initiation and progression	Review	De Craene B (15)	*Nature Reviews Cancer*	A review of EMT regulating cancer occurrence and progression	69.8	Q1	27
2010	Integrated Genomic Analysis Identifies Clinically Relevant Subtypes of Glioblastoma Characterized by Abnormalities in PDGFRA, IDH1, EGFR, and NF1	Article	Verhaak RGW (16)	*Cancer Cell*	A landmark article bringing related research into transcriptomic and genomic dimensions	38.585	Q1	27

JCR, Journal Citation Reports; EMT, epithelial–mesenchymal transition; GBM, glioblastoma.

### 3.4 Authors

The analysis of the research authors was from a VOS viewer (**Table S1** and **Figure 3B**). Among the thousands of authors, there were only two authors who have published more than 10 articles, namely, Qi SongTao (n=16) and Lei Wang (n=11). The few productive authors indicated that the field of EMT in gliomas is a relatively new and focused topic. On the list of top 10 cited authors, Nakano Ichiro had the highest citations (581). Wang Maode (432), Qi SongTao (367), and Wang Lei (331) were also highly cited. Similarly, the list of top 10 co-cited authors showed that Stupp R had the highest co-citations (253), indicating his influence in this field. Other highly co-cited authors include Thiery Jean Paul (193), David N Louis (183), and Ulf Dietrich Kahlert (170). In addition, the visualization of co-cited authors (**Figure 3B**) represented some co-citation relationships, like Stupp R/Louis DN and Kahlert UD/Thiery JP/Kalluri R.

### 3.5 Keywords and hot topics

The density visualization of author keywords from the VOS viewer (**Figure S3**) showed the most frequent keywords in this field. There were many cell factors and cellular activities connected with these high-density keywords. Through further data classification, three clusters of high-frequency keywords were obtained (**Table 2**). In the Molecule part, there were many signal molecules including β-catenin, Akt, Zeb1, STAT3, PI3K, Gli1, TGF-β, long non-coding RNA (lncRNA), microRNA (miRNA), and E-cadherin. In the Cancer and Treatment part, there were other cancers like breast cancer, gastric cancer, and treatment-related words including prognosis, temozolomide, and chemoresistance. In the Activity and State part, there were some cellular activities in addition to EMT including invasion, migration, proliferation, metastasis, apoptosis, and autophagy, and some cell states including hypoxia and stemness.

**Table 2:** The top 10 molecule, disease and treatment, and activity and state words in the author keywords.

Rank	Molecule	Count	Cancers and treatment	Count	Activity and state	Count
1	Beta-catenin	31	Glioma	336	EMT	415
2	Akt	26	Glioblastoma	285	Invasion	185
3	LncRNA	24	Prognosis	55	Migration	119
4	MiRNA	23	Temozolomide	38	Proliferation	102
5	Zeb1	21	Breast cancer	22	Metastasis	76
6	STAT3	20	Chemoresistance	21	Cancer stem cell	37
7	PI3K	20	Gastric cancer	16	Apoptosis	37
8	E-cadherin	19	Cancer	14	Hypoxia	25
9	Gli1	19	Hepatocellular carcinoma	14	Autophagy	19
10	TGF-beta	19	Colorectal cancer	13	Stemness	17

EMT, epithelial–mesenchymal transition; lncRNA, long non-coding RNA; miRNA, microRNA.

**Figure 4:**
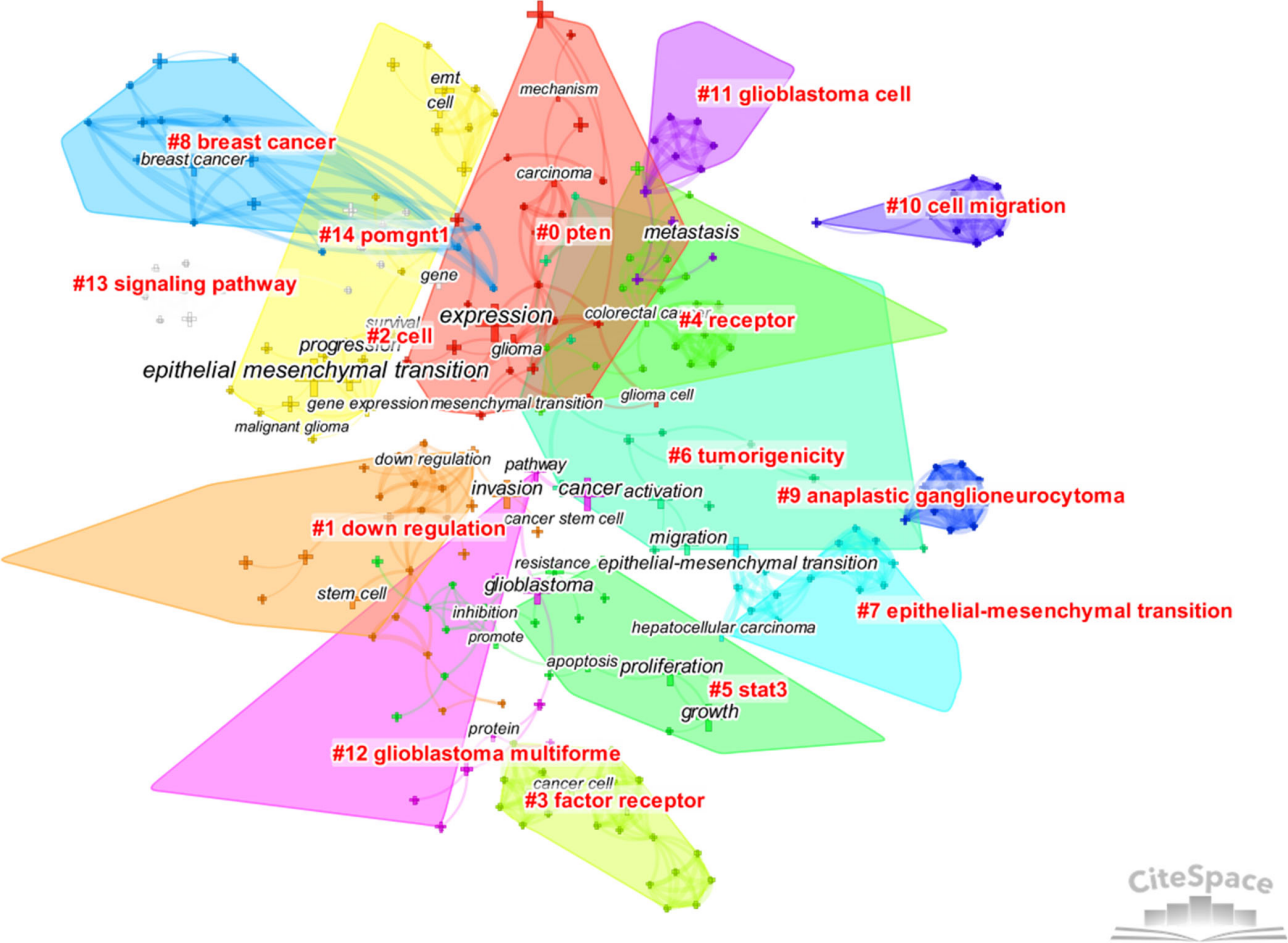
The cluster map of keywords. Pruning functions included pathfinder, pruning sliced networks, and pruning the merged networks. LLR algorithm was used.

CiteSpace was used to analyze the clusters of keywords. There were 15 clusters in total (**Figure 4**), and detailed information about the leading six clusters (#pten, #down regulation, #cell, #factor receptor, #receptor, and #stat3) is provided in **Table S2**.

From the analysis of keywords, the main hot topics of this field can be concluded as follows: 1) the characteristics of EMT in gliomas and its connection with other epithelial tumors, 2) the complex mechanisms regulating EMT in gliomas, and 3) the relationship between EMT and other cellular activities or states.

### 3.6 Research trends

**Figure 5:**
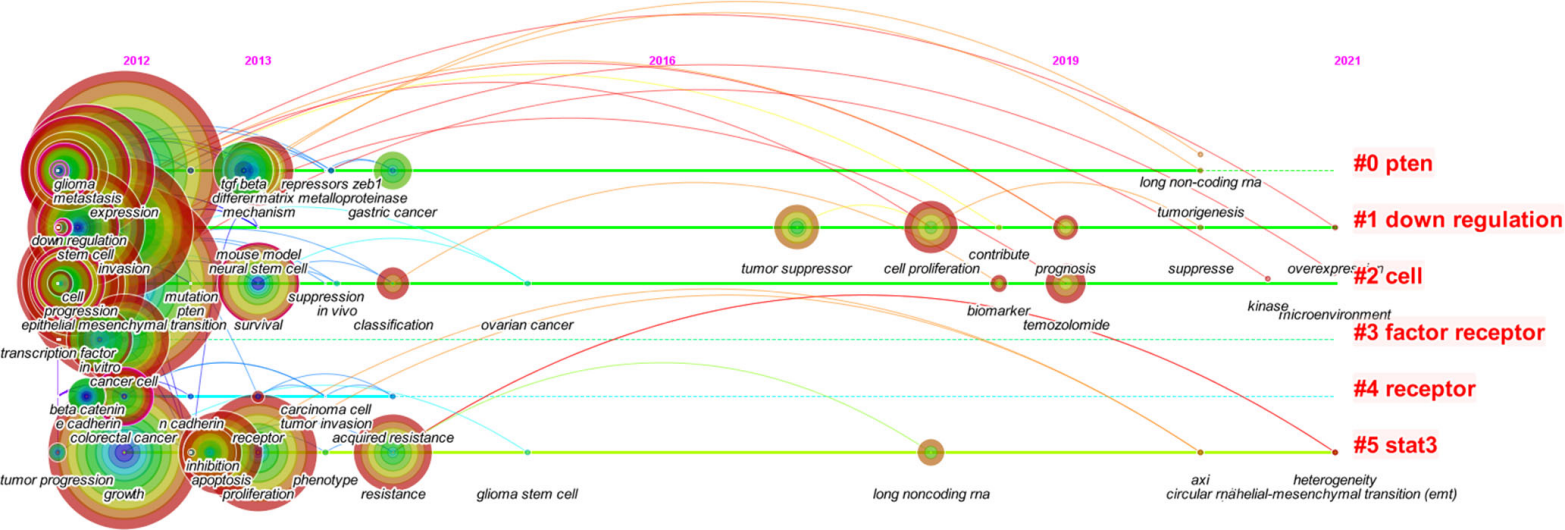
The timeline view of keywords in the six main clusters from 2012 to 2021.

**Figure 6:**
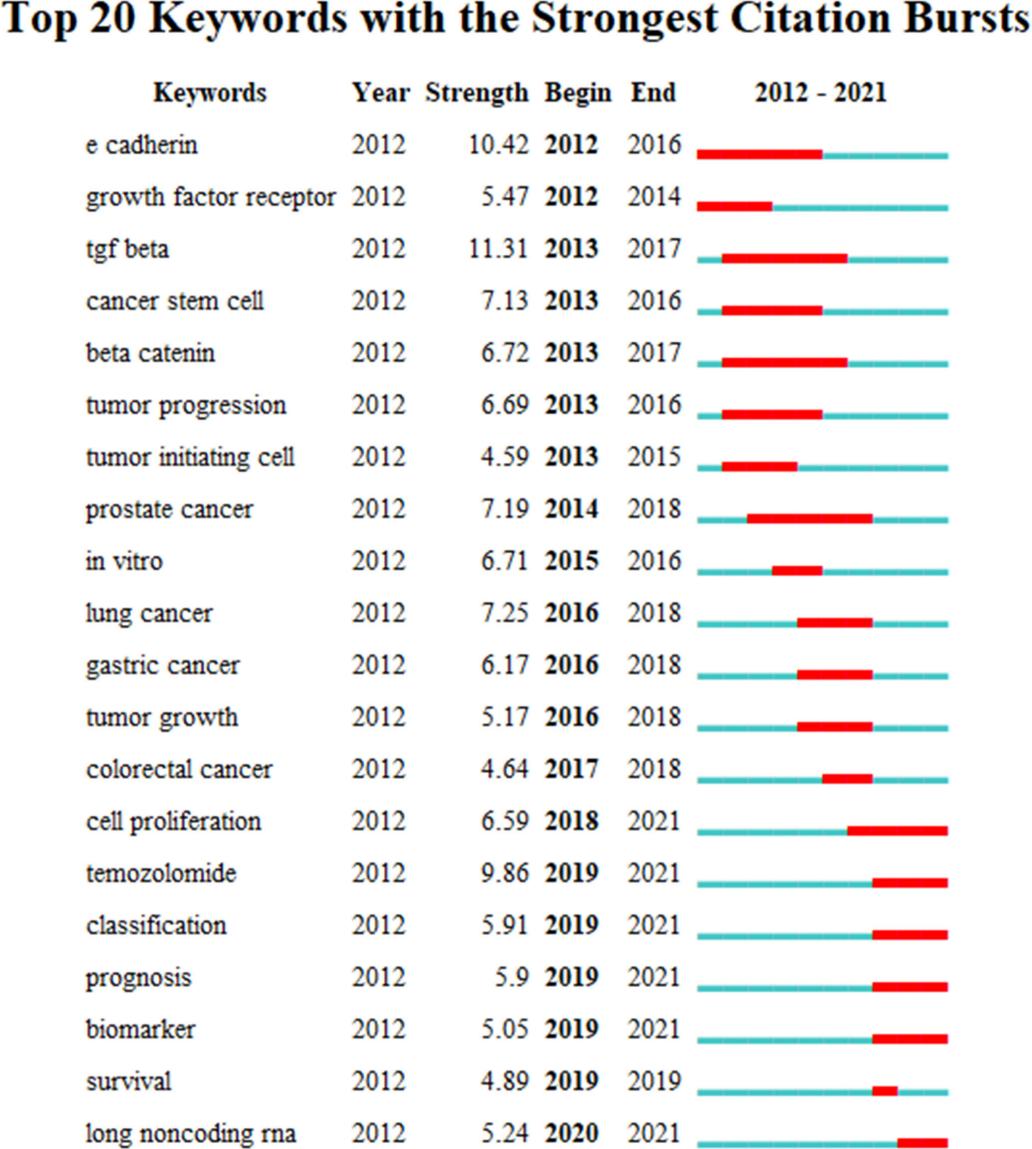
The top 20 keywords with the strongest citation bursts.

The burst analysis (**Figure 5**) and the timeline view (**Figure 6**) of keywords were performed in CiteSpace to understand the research trends in this field. Burst analysis of keywords was used to obtain the rapidly growing keywords in a short period, which can help understand the research trends in a specific field (7). In the top 20 keywords with the strongest bursts, 14 keywords were before 2018 such as E-cadherin, growth factor receptor, TGF-beta, cancer stem cell, beta-catenin, tumor progression, and tumor-initiating cell. After 2019 (including 2019), the six keywords were temozolomide, classification, prognosis, biomarker, survival, and long non-coding RNA. TGF-beta, E-cadherin, and temozolomide were the three strongest keywords. TGF-beta, E-cadherin, and beta-catenin had the longest durations (5 years). In addition, cell proliferation, temozolomide, classification, prognosis, biomarker, and lncRNA might continue to have strong bursts in the future.

In the timeline view of keywords, there were two major progression stages: one was before 2014 and the other was in 2017–2019. Moreover, the timeline view also displayed some new keywords in 2019–2021, like lncRNA, tumorigenesis, kinase, suppress, overexpression, microenvironment, circular RNA (circRNA), and heterogeneity.

Based on the burst analysis and the timeline view of keywords, it was found that cellular and molecular mechanisms of EMT in gliomas were widely studied in the early stage, and the hot topics extended to the treatment aspects in addition to the continuous exploration of regulatory mechanisms such as non-coding RNAs in recent years.

## 4 Discussion

The scientometric analysis provided us with the current research status, hot topics, and emerging trends of EMT in gliomas. According to this analysis, a summary of hot topics and emerging trends of EMT in gliomas is necessary.

### 4.1 The characteristics of epithelial–mesenchymal transition in gliomas and its connection with other epithelial tumors

Glioma cells possibly undergo EMT since they originate from glial cells that differentiate from neuroectoderm. However, research on EMT in gliomas started rather late as compared with other cancers. In 2010, a genome analysis classified GBM into four types, proneural, neural, classical, and mesenchymal subtypes, which laid the foundation for the following research (16). EMT in gliomas has many similarities in the cellular and molecular aspects when compared with other cancers. When EMT occurs in glioma cells, glioma cells express higher EMT markers including N-cadherin, MMPs, and transcription factors like Twists, Zebs, and Snails. These molecular changes will result in cellular changes like cytoskeletal organization, loss of cell–cell contact, and degradation of the basement membrane and finally enhance invasion, migration, and metastasis of gliomas (3).

However, there are also some typical differences. When EMT occurs, glioma cells do not go through the cadherin switch (from E-cadherin to N-cadherin) because they may have been more mesenchymal than epithelial in the initial stage (2). Therefore, some scholars proposed using EMT-like or glial–mesenchymal transition to describe EMT in gliomas (3, 17).

### 4.2 The complex mechanisms regulating epithelial–mesenchymal transition in gliomas

**Figure 7:**
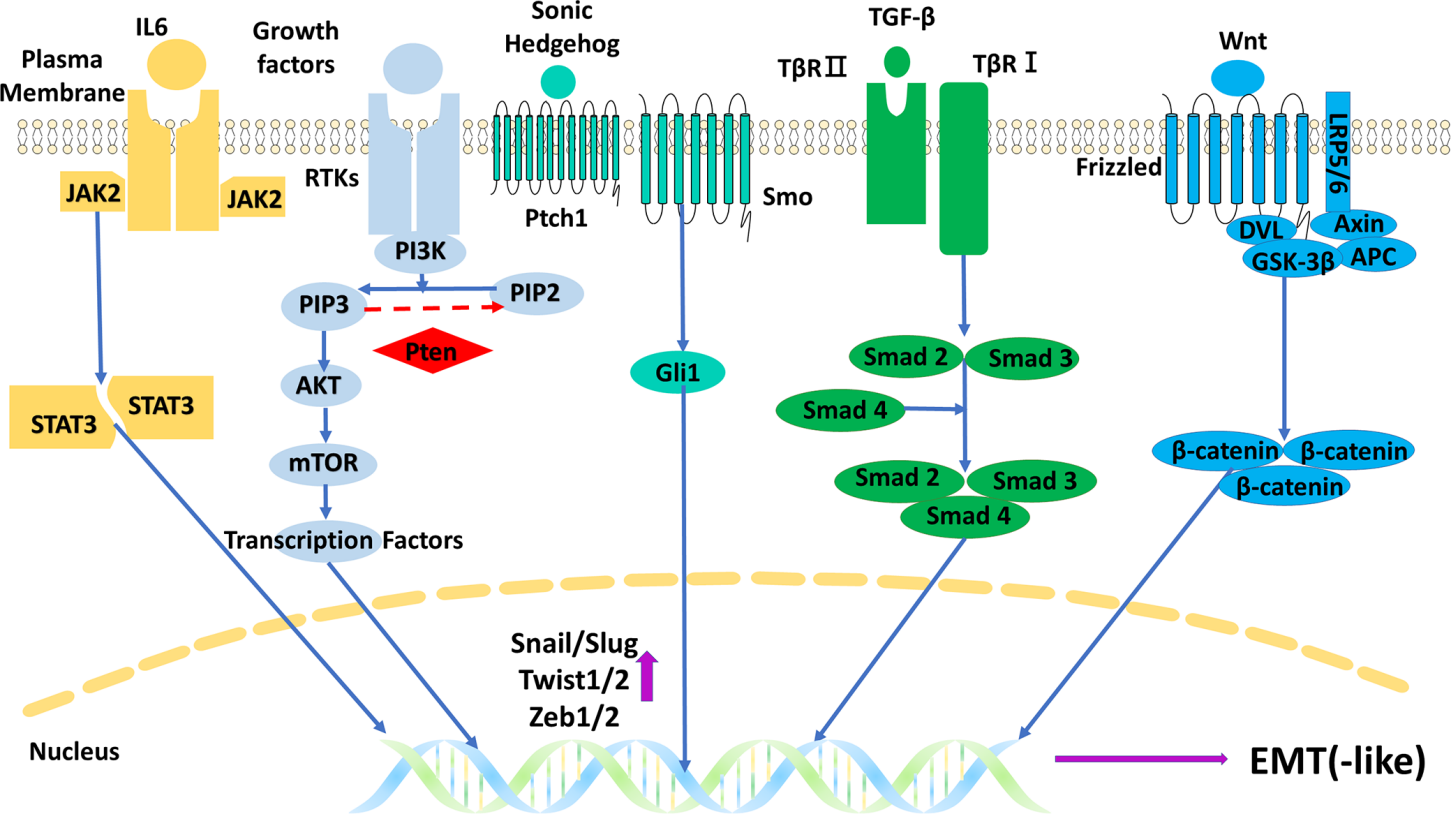
The main five signal pathways regulating EMT in gliomas. EMT, epithelial–mesenchymal transition.

**Figure 8:**
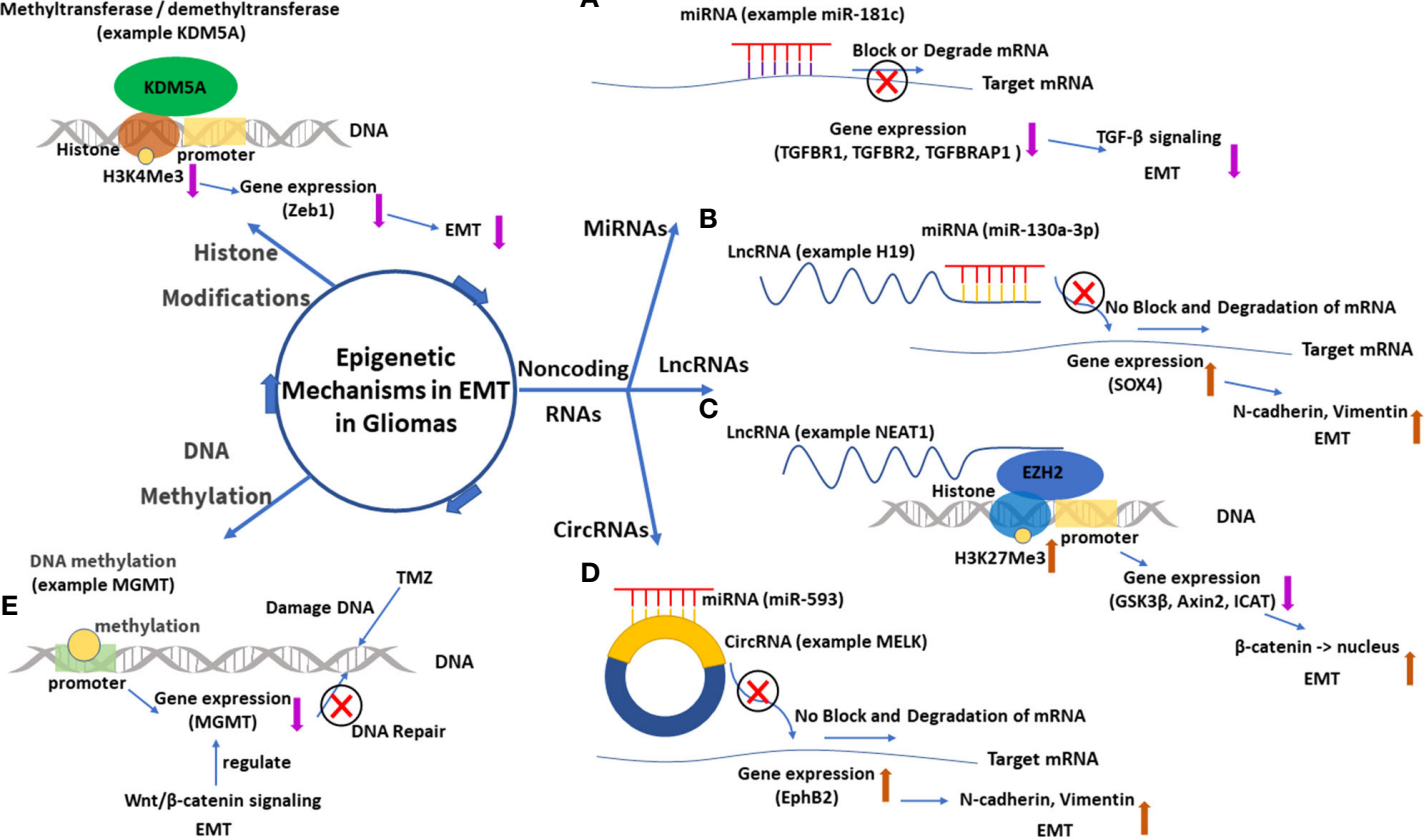
The main epigenetic mechanisms in EMT in gliomas. **(A)** MiRNA 181-c and its function of gene silence (TGFBR1, TGFBR2, and TGFBRAP1), which inhibits the TGF-β signal and suppresses EMT. **(B)** LncRNA H19 acts as a miRNA 130a-3p sponge, which enhances SOX4 expression and promotes EMT. **(C)** LncRNA NEAT1 downregulates expressions of GSK3β, Axin2, and ICAT by recruiting EZH2, which catalyzes H3K27Me3. The downregulated GSK3β and Axin2 then promote the nuclear transport of β-catenin, which enhances EMT. **(D)** CircRNA MELK acts as a miRNA 593 sponge, which enhances EphB2 expression and promotes EMT. **(E)** Methylation of the promoter of the MGMT gene downregulates MGMT expression and enhances the effect of TMZ. EMT can regulate MGMT expression through the Wnt/β-catenin signal pathway. **(F)** Histone demethyltransferase KDM5A decreases the content of H3K4Me3, which downregulates Zeb1 and suppresses EMT. EMT, epithelial–mesenchymal transition; lncRNA, long non-coding RNA; circRNA, circular RNA; miRNA, microRNA.

The regulatory mechanisms are the core part of research on EMT in gliomas, and understanding these mechanisms is the foundation of further research such as target inhibitions. There are some important molecules and important regulatory mechanisms for EMT in gliomas (**Figures 7**, **8**), which can be revealed in the analysis of the keywords.

#### 4.2.1 Epidermal growth factor receptor

Receptors play significant roles in regulating EMT in gliomas, which could be also indicated by its two occurrences in the cluster keywords. Among the various receptors, epidermal growth factor receptor (EGFR) has been researched the most. EGFR gene has the highest amplification frequency (59.4%) of all receptor tyrosine kinases (RTKs) in GBM, and the frequency is up to 95% in the classical subtype, which has the worst prognosis (18, 19). The overexpression of EGFR will excessively activate EGFR-dependent signal pathways including PI3K/Akt, JAK2/STAT3, and Ras/MAPK in the course of EMT in gliomas (3). In addition to the amplification, rearrangement of the EGFR gene and its encoded protein EGFR variants are also common in gliomas. EGFR vIII (EGFR variant III, deletion of exons 2 to 7) is the commonest variant, occurring in 55% of GBM (20). This variant is constitutively activated owing to the loss of the ligand-binding domain and has different roles compared with EGFR amplification in gliomas. It is believed that occurrences of EGFR vIII reflect an increase in glioma heterogeneity, which is more common in advanced gliomas and can promote angiogenesis (20, 21), whereas wild-type EGFR gene amplification occurs in all stages of gliomas and can greatly promote invasion and proliferation instead of having effects on angiogenesis (18). It seems that wild-type EGFR amplification is more important in regulating EMT in gliomas, but the high heterogeneity and angiogenesis mediated by EGFR vIII are also beneficial to EMT. EMT is beneficial to the high heterogeneity and angiogenesis in turn. Moreover, inhibitions of EGFR are widely explored in gliomas like EGFR tyrosine kinase inhibitors and monoclonal antibodies, though few have excellent effects (22–24). Hopefully, some novel therapies emerged in recent years, like chimeric antigen receptor-T cell (CAR-T) therapy targeted on EGFR vIII and nanoparticle delivery of EGFR inhibitors, but their real effectiveness in gliomas is yet to be explored (22, 23).

#### 4.2.2 c-Met

c-Met is the third most commonly amplified RTK in GBM, and its expression is related to GBM subtypes (25). The mesenchymal and proneural subtypes often highly express c-Met. The higher expression of c-Met also often occurs in GBM recurrences with a worse prognosis (26). It was found that the expression of c-Met seems to have an antagonistic relationship with the expressions of EGFR and vascular EGFR (VEGFR). When c-Met is upregulated, it often couples with the downregulations of EGFR and VEGFR, and the reverse is true (27, 28). c-Met is more associated with a stronger invasion of gliomas by inducing stronger EMT compared with EGFR and VEGFR (27, 28). This antagonistic relationship may indicate the necessity of combined c-Met inhibitions when considering giving anti-angiogenesis or anti-EGFR therapies for glioma patients.

#### 4.2.3 TGF-β

TGF-β was the strongest burst keyword, indicating its popular role in the research of EMT in gliomas. Although TGF-β can act as an EMT suppressor in premalignant stages of gliomas (29), the main regulatory mechanism for TGF-β is to promote EMT through the TGF-β/Smad pathway in progression stages (30, 31). Moreover, TGF-β can also promote EMT through some Smad-independent ways like PI3K-Akt-mTOR and Ras/MAPK pathways (30). Inhibitions of TGF-β and its pathways are widely researched, like antisense oligonucleotides (AONs) of TGF-β messenger RNA (mRNA), monoclonal antibodies of TGF-β, and TGF-β receptor kinase inhibitors (29, 32).

#### 4.2.4 Wnt/β-catenin

As the keyword with the highest frequency in the Molecule part, β-catenin and the Wnt/β-catenin pathway have been researched well in EMT in gliomas. The main regulatory mechanism is the canonical Wnt/β-catenin pathway. The pathway includes Wnt, Frizzled receptor, co-receptor LRP5/6 (low-density lipoprotein receptor-related protein 5/6), complex (degrading β-catenin in the absence of Wnt) comprising APC (adenomatous polyposis coli), GSK3β (glycogen synthase kinase-3β) and Axin, and β-catenin with its target genes like Snails, Zebs, and Twists (3, 33). Moreover, both β-catenin and Wnt have independent ways to promote EMT. β-Catenin can act as the binding protein of cadherin to regulate cell–cell adhesion, and this binding relationship can be disrupted through the phosphorylation of β-catenin triggered by some growth factors (33). Wnt5a and its β-catenin-independent downstream can elevate the expression of MMP2 to promote EMT (34). There are many targets on the Wnt/β-catenin pathway, but their related inhibitions often fail. The reasons are multidimensional, like the important role they play in the normal tissues and the existence of the blood–brain barrier (34, 35).

#### 4.2.5 PI3K/Akt and PTEN

PI3K/Akt pathway is another key signal pathway regulating EMT in gliomas. In this pathway, the activated PI3K transforms PIP2 (phosphatidylinositol-4,5-bisphosphate) to PIP3 (phosphatidylinositol-3,4,5-triphosphate), and then PIP3 recruits and activates Akt, and Akt phosphorylates and activates its downstream like mTOR (36). PTEN protein is a tumor suppressor that can convert PIP3 to PIP2 and inhibit this pathway. Many glioma patients detect loss or mutation or promotor methylation of the PTEN gene, which results in the overactivation of the PI3K/Akt pathway (37). Research showed that PI3K/Akt pathway has a high possibility to be activated in gliomas, with potential mechanisms including PTEN gene change, EGFR gene amplification, and non-coding RNAs (36, 38). In the aspect of targeted inhibitions, inhibiting several targets instead of one may obtain better results, like targeting EGFR and PI3K/mTOR simultaneously (36).

#### 4.2.6 STAT3

As the cluster keyword, STAT3 and its related pathways like JAK2/STAT3 pathway are widely researched in gliomas. JAK2/STAT3 pathway can be activated by IL-6 and some growth factors. The activated STAT3 can be transported to the nucleus to upregulate expressions of MMP2 and Twists (39, 40). The expression of IL-6 often remains at a high level in gliomas, especially GBM, which results in the overactivation of the JAK2/STAT3 pathway and induces stronger EMT (39). Interestingly, STAT3 can also take part in a tumor-suppressive pathway, LIFRβ (leukemia inhibitory factor receptor β)/STAT3 pathway. However, this pathway is often silenced in gliomas since the high level of PIP3 (related to PTEN loss and PI3K/Akt overactivation) can inhibit the activation of LIFRβ (41). Moreover, the potential targets of the JAK2/STAT3 pathway and their related inhibitions are also popularly researched, including the receptor–ligand interaction, the kinase activity of the components, and the activity/dimerization/transportation of STAT3(s) (40, 42).

#### 4.2.7 Hedgehog/Gli1

Hedgehog/Gli1 pathways are often found overactivated in GBM, and Sonic Hedgehog (SHH) is the most researched hedgehog in EMT in gliomas (43). SHH can activate Gli1 through binding to receptor Patched1 (PTCH1) and activating receptor smoothened (SMO). Then Gli1 translocates to the nucleus to upregulate the expression of EMT markers like MMP2/9 and Twists (43). Some variants of Gli1 like truncated Gli1 (tGli1) are regarded as an enhanced Gli1 to induce EMT and stronger invasion in GBM, which may act as a target for inhibitions (44).

#### 4.2.8 MicroRNAs

Non-coding RNAs are the main research topic for epigenetic regulation in EMT in gliomas. Among several kinds of non-coding RNAs, research on miRNAs was the earliest. MiRNAs are single-stranded non-coding RNAs with a length of 20–23 nucleotides. The roles of miRNAs have been researched well, which mainly regulate the translation by binding and interacting with the targeted mRNA (45, 46). For example, miR-181c targets TGFBR1, TGFBR2, and TGFBRAP1 mRNAs to inhibit their expressions. EMT is inhibited by the downregulation of TGF-β signaling (45).

#### 4.2.9 Long non-coding RNAs

LncRNAs are another type of non-coding RNAs with more than 200 nucleotides that are transcribed mainly by RNA polymerase II. These longer non-coding RNAs have gained much attention in recent years because of their complex and significant roles in regulating molecular changes and cellular activities. However, compared with miRNAs, the definite roles of lncRNAs are yet to be explored. In most research on EMT in gliomas, lncRNAs act as miRNA sponges or competing endogenous RNAs (ceRNAs) that lncRNA can modulate miRNA availability by vying with mRNAs to bind miRNA (47, 48). This specific interaction model is called mRNA–miRNA–lncRNA network or miRNA/lncRNA axis. LncRNA H19 is a typical example of this interaction model, serving as a sponge of MiR-130a-3p. When MiR-130a-3p is sponged, the expression of its target gene SOX4 is upregulated, which is beneficial to EMT by enhancing N-cadherin and vimentin (49). Another important regulatory mechanism for lncRNAs in the cytoplasm is that they directly bind to proteins and modulate their functions through phosphorylation or ubiquitination, which is especially important to some molecules in the signal pathways (47, 48, 50). In the nucleus, some lncRNAs can also directly or indirectly regulate genome activity, transcription, or post-transcription modifications (47, 48, 51). For example, lncRNA NEAT1 can serve as a scaffold and recruit EZH2 (a chromosome modification enzyme) to silence some genes (GSK3β, Axin2, and ICAT), which can increase β-catenin nuclear transport and enhance EMT. As the analysis of keywords showed, lncRNAs seemed to have become one of the hottest topics in EMT in gliomas. It is believed that with a deeper exploration of lncRNAs, more related therapies will be proposed and promoted.

#### 4.2.10 Circular RNAs

CircRNAs are covalently closed single-stranded RNAs that are typically found in the cytoplasm of eukaryotic cells. These special RNAs play similar roles as lncRNAs, such as the miRNA sponge effect, interaction with RNA binding proteins, and participation in protein translation (52). Research on circRNAs regulating EMT in gliomas is insufficient, and the current research mainly concentrates on the miRNA sponge effect (53). CircRNA MELK acts as a sponge of miR-593 that targets EphB2 gene. When MELK is upregulated in GBM, EphB2 will be upregulated and promote EMT and glioma stem cell (GSC) maintenance (53). CircRNA PIP5K1A has a similar interaction model in that PIP5K1A sponges miR-515-5p to upregulate and activate the TCF12-PI3K/Akt axis to promote EMT (54). Moreover, it is visible that the regulatory networks of four RNAs (miRNAs, lncRNAs, circRNAs, and mRNAs) are gradually built up, which will surely give us more new insights into EMT in gliomas.

#### 4.2.11 Other epigenetic mechanisms

In addition to the three non-coding RNAs, DNA methylation and histone modifications are also involved in the epigenetic mechanisms for EMT in gliomas. For DNA methylation in gliomas, a known example is the gene *O* ^6^-methylguanine-DNA methyltransferase (MGMT). When the promoter of gene MGMT is methylated, its encoded protein MGMT will be downregulated. This will surely impair the function of repairing DNA for MGMT, which is beneficial to the therapeutic effect of temozolomide (TMZ) for glioma patients (55). Moreover, it was found that the expression of MGMT can be regulated by EMT through the Wnt/β-catenin pathway, which supports the use of inhibitors of Wnt/β-catenin signaling and temozolomide in combination for patients expressing high levels of MGMT (55, 56).

The main histone modification for EMT in gliomas is methylation or demethylation. An example of histone methylation is the gene of lncRNA LINC-PINT. When Twist1 is methylated by SETD6 (a methyltransferase), it will increase the occupancy of EZH2 (a histone lysine methyltransferase) and the catalysis of the repressive H3K27Me3 (histone H3 lysine 27 trimethylation) mark at the locus of lncRNA LINC-PINT (a tumor repressor in gliomas). The downregulated LINC-PINT will reduce cell adhesion and enhance cell migration and EMT (57). In addition to methylation, histone demethylation is also important for EMT in gliomas. KDM6B, a histone lysine demethyltransferase, can decrease the content of H3K27Me3 in the promoter region of Snai1 gene. The upregulated Snai1 will promote EMT and cell migration (58). Another histone lysine demethyltransferase KDM5A shows a similar role but a different result in EMT in gliomas. KDM5A can decrease the content of H3K4Me3 (histone H3 lysine 4 trimethylation) in the promoter region of Zeb1 gene and then decrease Zeb1 and inhibit EMT (59). The different results are made by the different interaction sites in that methylation at H3K4 is usually associated with gene activation, whereas methylation at H3K27 is associated with the inhibition of gene transcription (59).

### 4.3 The relationship between epithelial–mesenchymal transition and other cellular activities or states

EMT is not an independent cellular activity in gliomas. It has complex relationships with other cellular activities or states like proliferation, stemness, hypoxia, and autophagy, which were indicated in the analysis of the keyword.

#### 4.3.1 Proliferation and stemness

Proliferation is often accompanied by EMT because the two processes often depend on the same signal pathways, and some cancer cells undergoing EMT can obtain stemness or become cancer stem cells (60). Cancer stem cells refer to a subset of cancer cells with the ability of self-renewal and multipotency. These cells usually express some specific stem markers, like CD-133, OLIG2, and SOX2, in gliomas (3). It is widely accepted that EMT and the features of GSCs (stemness) are co-regulated in the invasion and metastasis of gliomas. Namely, the downregulation or upregulation of stem markers contributes to the downregulation or upregulation of EMT markers, and vice versa (4). Based on this co-regulatory mechanism, more and more biomarkers and targets of EMT in gliomas have been explored in recent years (61). Moreover, the proneural subtype of GBM is characterized by stemness, and in the recurrences, GBM often switches to the mesenchymal subtype, which can also indicate the intimate connection between stemness and EMT in gliomas (16).

#### 4.3.2 Hypoxia

Hypoxia occurs in certain regions of gliomas especially high-grade gliomas (HGGs), because of excessive oxygen consumption by quick cell proliferation or anti-angiogenic therapy. To better adapt to the hypoxia environment, cancer cells will usually become more invasive through EMT. In this regulatory process, HIF1 (hypoxia-induced factor 1) and some EMT markers like Snails and Twists are key factors. Some mechanisms for hypoxia-inducing EMT in gliomas have been recognized; for example, VEGF inhibitor therapy will activate c-Met and upregulate Snail and N-cadherin (28), and HIF-1α can upregulate Zeb2, which will downregulate EphrinB2, an invasion-inhibitory receptor in gliomas (62). The intimate connections between hypoxia and EMT indicate that target therapies on both EMT and angiogenesis may be a necessary choice for glioma patients.

#### 4.3.3 Autophagy

In many ways like hypoxia, nutrient starvation can contribute to autophagy (mainly macroautophagy), and autophagy can be mediated by mTOR and its upstream signal pathways like PI3K/Akt, and its targeted autophagy-related (ATG) genes (63). The connection between autophagy and EMT was proposed recently (64), and it remains controversial in gliomas (63). Some believe that autophagy acts as tumor-suppressive signals and inhibits EMT by destabilizing EMT-related molecules in the early stages of gliomas, and in progression stages, cancer cells with EMT require autophagy to survive the environmental and metabolic stressful conditions (63, 64). Others hold the belief that the influences of autophagy on EMT are mediated by different inducers instead of tumor stages. On the one hand, autophagy induced by nutrient starvation or by inhibition of mTOR impairs EMT for glioma cells. On the other hand, autophagy induced by drugs like temozolomide plus low glucose, or TGF-β, or the overexpression of ATG can promote EMT (63, 65). These two different opinions show the complex mechanisms between autophagy and EMT need to be explored further and deeper.

#### 4.3.4 Chemoresistance

The most frequent and effective chemotherapy drug for gliomas is TMZ, a DNA alkylating agent that can readily penetrate the blood–brain barrier and play its role. However, TMZ resistance often occurs. An important contributor to this chemoresistance is EMT. In several subtypes of GBM, the proneural subtype has a good reaction to TMZ as compared with other subtypes. However, when the proneural subtype recurs, it often undergoes a transition to the mesenchymal subtype, which has strong resistance to TMZ. This transition of subtypes indicates that EMT can probably mediate TMZ resistance in some ways (16, 66). MGMT, a DNA damage repair protein that removes the guanine-alkyl group, is an important contributor to TMZ resistance (67). It was discovered that the expression of MGMT can be regulated by EMT through the Wnt/β-catenin pathway (55, 56). Moreover, autophagy-induced EMT may be another potential mechanism resulting in chemoresistance (68). Based on the analysis of keywords and the unique role of TMZ in treating gliomas, it is believed that more research should be conducted to offset the resistance of TMZ in addition to research on targeted therapies.

## 5 Conclusions

The scientometric analysis of 1,216 publications related to EMT in gliomas shows that the field developed fast and received continuous attention in the last decade. The hot topics of EMT in gliomas mainly focused on the cellular and molecular mechanisms, and EMT-related targeted therapies and non-coding RNAs have also been widely explored in recent years. Based on the scientometric analysis, the review of three hot topics was made to help those who specialize in gliomas better understand the current research status of this field.

## Author contributions

JC and XH conceived the study and performed the critical revision of the manuscript. YX designed the study, performed the statistical analyses, and drafted the manuscript. MH, ZS, FY, and JL designed the study and wrote the manuscript. YX and MH performed the article retrieval and data interpretation. JC, XH, JL, and HW helped modify the manuscript. All authors contributed to the article and approved the submitted version.

## Funding

This work was supported by the Natural Science Foundation of Jilin Province, China (Grant No. 20200201491JC), and the Health Planning Commission of Jilin Province, China (Grant No. 2018Q026).

## Acknowledgments

We greatly appreciate Dr. Yongfeng Liu for modifying the language of this article.

## Conflict of interest

The authors declare that the research was conducted in the absence of any commercial or financial relationships that could be construed as a potential conflict of interest.

## Publisher’s note

All claims expressed in this article are solely those of the authors and do not necessarily represent those of their affiliated organizations, or those of the publisher, the editors and the reviewers. Any product that may be evaluated in this article, or claim that may be made by its manufacturer, is not guaranteed or endorsed by the publisher.

## Abbreviations

EMT, epithelial–mesenchymal transition; GBM, glioblastoma; MMP, matrix metalloproteinase; WoSCC, Web of Science Core Collection; SCIE, Science Citation Index Expanded; MeSH, Medical Subject Heading; TS, topic subject; WoS, Web of Science; lncRNA, long non-coding RNA; miRNA, microRNA; circRNA, circular RNA; RTK, receptor tyrosine kinase; EGFR vIII, EGFR variant III; ceRNA, competing endogenous RNA; MGMT, *O* ^6^-methylguanine-DNA methyltransferase; TMZ, temozolomide; H3K27Me3, histone H3 lysine 27 trimethylation; H3K4Me3, histone H3 lysine 4 trimethylation; GSC, glioma stem cell; HIF1, hypoxia-induced factor 1; ATG, autophagy-related gene.
