# Supplementary material for: Emerging trends and research foci of epithelial–mesenchymal transition in gliomas: A scientometric analysis and review
Source: Front Oncol. 2022 Oct 20;12:1015236. doi: 10.3389/fonc.2022.1015236 (PMC9632964; doi:10.3389/fonc.2022.1015236)
Supplement: Supplementary file 1 [file DataSheet_1.docx]

**Supplementary Material**

**1 Supplementary Figures and Tables**

**1.1 Supplementary Figures**


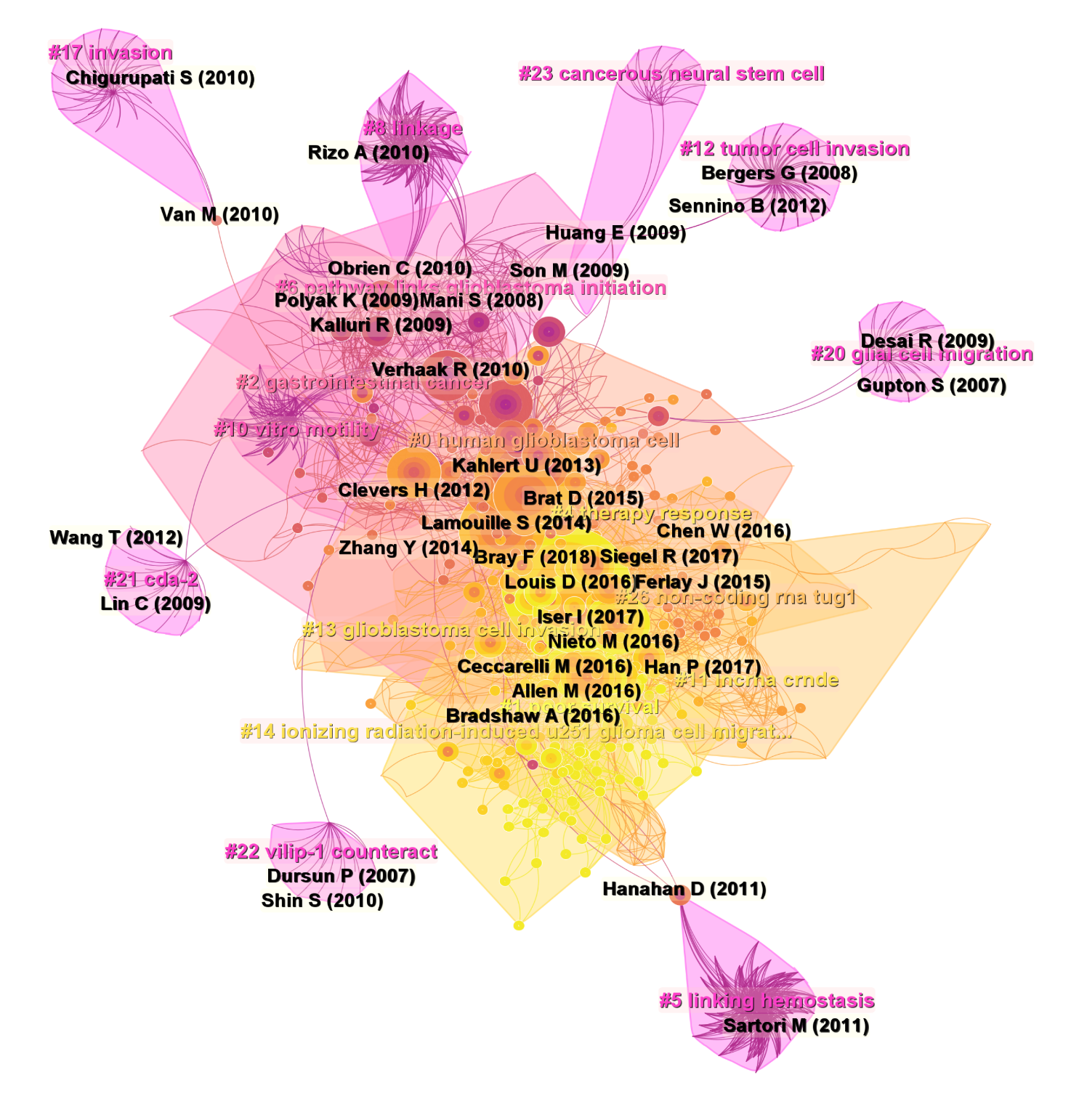


**Figure S1**. The cluster map of highly co-cited references from Citespace. The names of clusters: #0 human glioblastoma cell, #1poor survival, #2gastrointestinal cancer, #4therapy response, #5linking hemostasis, #6pathway links glioblastoma initiation, #8linkage, #10vitro motility, #11lncrna crnde, #12tumor cell invasion, #13glioblastoma cell invasion, #14ionizing radiation-induced u251 glioma cell migration, #17invasion, #20glial cell migration, #21cda-2, #22vilip-1 counteract, #23cancerous neural stem cell, #26non-coding rna tug1.


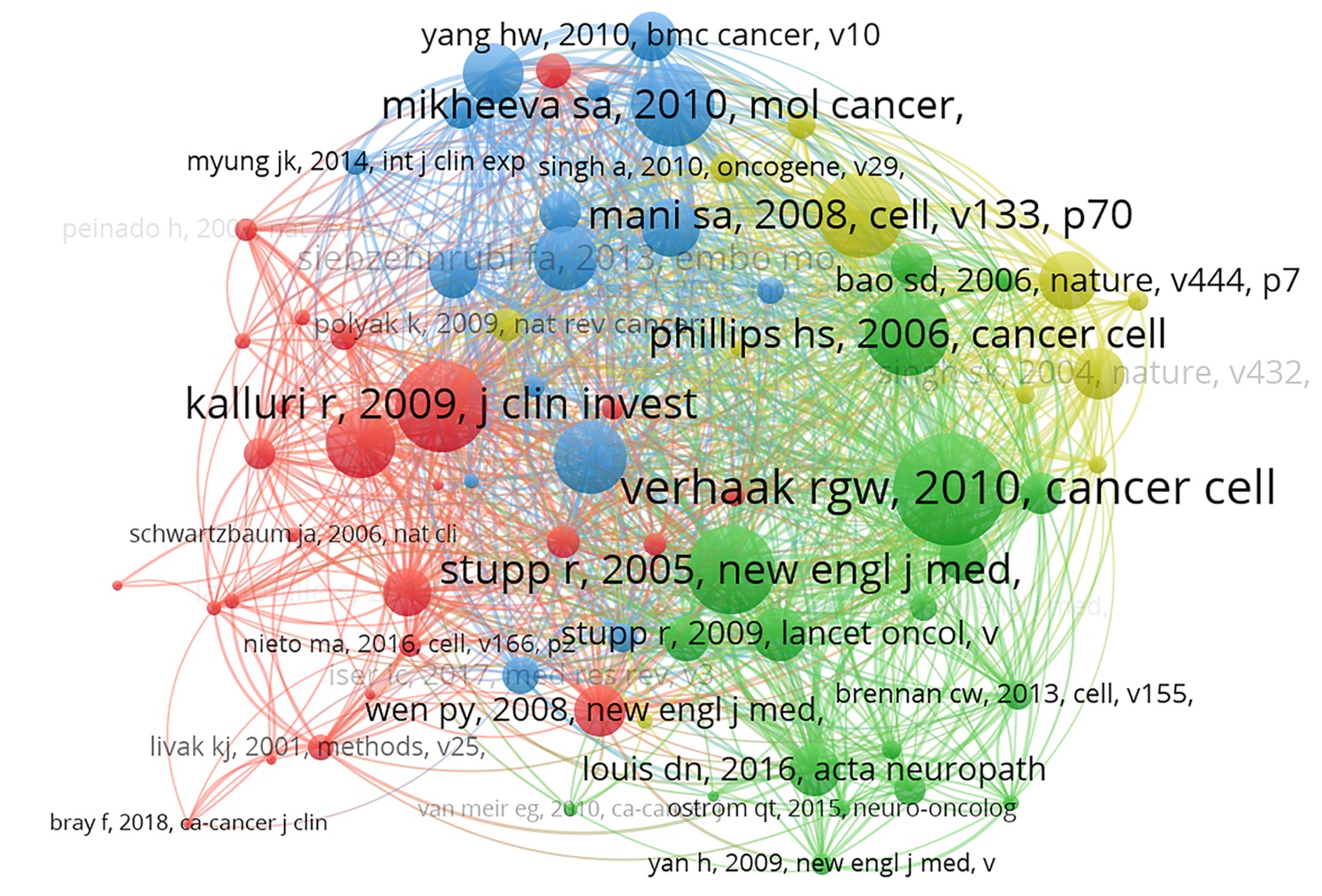


**Figure S2.** The co-occurrence map of highly co-cited references from VOS viewer.


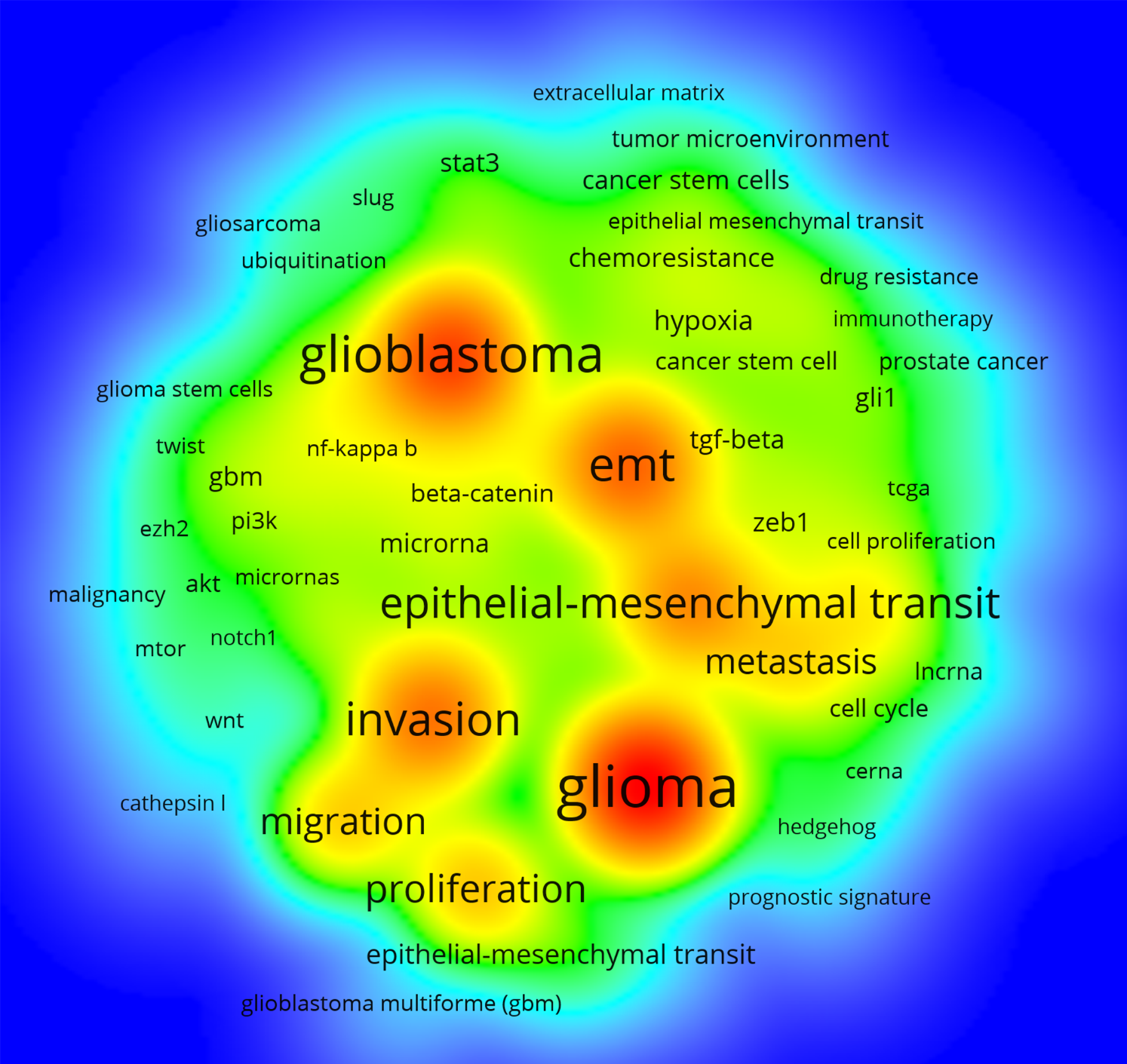


**Figure S3.** The density visualization of the author keywords. Keywords with the background color Red represent the high density and importance.

**1.2 Supplementary Tables**

**Table S1.** The two lists of the top 10 cited authors and the top 10 co-cited authors.

| The top 10 cited authors | | | The top 10 co-cited authors | | |
| --- | --- | --- | --- | --- | --- |
| Author | Nationality | Citations | Author | Nationality | Co-Citations |
| Nakano Ichiro | USA | 581 | Stupp Roger | USA | 253 |
| Wang Maode | China | 432 | Thiery Jean Paul | USA | 193 |
| Qi Songtao | China | 367 | David N Louis | USA | 183 |
| Wang Lei | China | 331 | Ulf Dietrich Kahlert | USA | 170 |
| Zhang Miqin | China | 223 | Kalluri Raghu | USA | 147 |
| Maciaczyk Jaroslaw | Germany | 223 | Quinn T Ostrom | USA | 118 |
| Long Hao | China | 211 | Verhaak RGW | USA | 115 |
| Song Ye | China | 204 | Zhang Y | China | 101 |
| Jiang Tao | China | 204 | Wang Y | China | 100 |
| Zhang Wei | China | 200 | Mani Sendurai A. | USA | 97 |

**Table S2.** The six leading clusters with all the keywords covered.

| Clusters | Keywords (in order of word frequency from high to low) |
| --- | --- |
| #pten  (26, S=0.867) | expression, metastasis, glioma, carcinoma, mesenchymal transition, mechanism, glioma cell, differentiation, tgf beta, gastric cancer, pancreatic cancer, transcription, cell invasion, tumorigenesis, long non-coding rna, repressors zeb1, matrix metalloproteinase, mir 200 family, involvement, ductal adenocarcinoma, transcription factor sp1, side population, human gastric cancer, cell lung cancer, caveolin 1 up regulation |
| #down regulation  (26, S=0.953) | invasion, stem cell, down regulation, cell proliferation, tumor suppressor, up regulation, prognosis, angiogenesis, snail, contribute, rna, overexpression, suppresse, beta, sonic hedgehog, mouse model, neural stem cell, disease, factor bearing microparticle, coagulation, tgf-beta 1, risk factor, recurrence, thrombosis, microvesicle, vegf |
| #cell  （23, S=0.968） | epithelial mesenchymal transition, progression, cell, emt, survival, gene expression, malignant glioma, microrna, temozolomide, classification, transition, biomarker, ovarian cancer, microenvironment, in vivo, kinase, profile, suppression, mutation, pten, human urine extract, promotes invasion, medulloblastoma |
| #factor receptor  (23, S=0.982) | cancer cell, in vitro, factor receptor, transcription factor, c6 glioma cell, endothelial growth factor, elevating agent, inducible factor i, keratinocyte migration, e cadherin expression, epithelial cell, fatty acid synthase, growth factor i, functional significance, met protooncogene, visinin like protein 1, transforming growth factor beta 1, glioblastoma cell, kappa b pathway, c met, tyrosine phosphatase 1b, valproic acid, antiangiogenic therapy |
| #receptor  (22, S=0.979) | colorectal cancer, e cadherin, beta catenin, receptor, n cadherin, acquired resistance, tumor invasion, carcinoma cell, wnt pathway, vascular endothelial cell, trans retinoic acid, tissue factor expression, tissue factor, procoagulant activity, plasminogen activator, negative regulator, molecular weight heparin, lentiviral vector, glioma tumorigenesis, factor promoter, epithelial-to-mesenchymal transition(emt), cancer stem cell niche |
| #stat3  (19, S=0.869) | growth, proliferation, apoptosis, resistance, inhibition, promote, long noncoding rna, tumor progression, phenotype, self renewal, heterogeneity, axi, circular rna, epithelial-mesenchymal transition (emt), glioma stem cell, invasiveness, colon, signature, intrahepatic cholangiocarcinoma |
